# Broadcasters’ expertise and consumers’ purchase intention: The roles of consumer trust and platform reputation

**DOI:** 10.3389/fpsyg.2022.1019050

**Published:** 2022-11-03

**Authors:** Jie Li, Ruyi Zheng, Haiqin Sun, Jiaoying Lu, Wenbo Ma

**Affiliations:** ^1^International Business School Suzhou, Xi’an Jiaotong-Liverpool University, Suzhou, China; ^2^Graduate Institute of Interpretation and Translation, Shanghai International Studies University, Shanghai, China

**Keywords:** broadcaster expertise, purchase intention, consumer trust, platform reputation, farmer-assisted live streaming

## Abstract

Following the outbreak of COVID-19, farmer-assisted live streaming has become a hot topic in China. In this manuscript, we explore the ways in which broadcaster and platform characteristics jointly influence consumers’ purchase intention. To examine our hypotheses, we distributed questionnaires to 261 Chinese consumers who viewed farmer-assisted live streaming. Correlational analyses, regression analyses, and confirmatory factor analyses were conducted to examine our hypotheses. The results show that broadcasters’ expertise is positively related to consumer trust and that platform reputation moderates this relationship. In addition, consumer trust mediates the positive relationship between broadcasters’ expertise and consumer purchase intention. Furthermore, the theoretical and practical implications of these findings are discussed.

## Introduction

Since 2020, the development of the Chinese consumer market has been hampered by the impact of the COVID-19 epidemic, and the sale of agricultural products has also suffered (Hung, 2021). Simultaneously, the effect of the epidemic has led to a significant change in terms of the rapid expansion of public shops and online shopping during the epidemic (Moon et al., 2021). Agricultural products have shifted from traditional offline consumption to online sales. In this context, live streaming media are popular due to their instant and high-intensity mode of interaction. Broadcasters recommend products to consumers by using live streaming to show products and introduce products simultaneously to promote consumers’ purchase behavior. As of June 2021, there were 638 million live internet users in China, an increase of 75.39 million over the pre-epidemic period; this total includes 264 million online e-commerce users (China Internet Network Information Center [CNNIC], 2021). Moreover, to sell agricultural products more effectively, the government also helps farmers sell agricultural products, with many local officials serving as broadcasters and promoting local produce to consumers *via* live streaming. Live streaming to provide agricultural assistance has made an essential contribution to the task of addressing issues pertaining to the development of the agricultural economy (Chen et al., 2021). Due to its enormous potential, this phenomenon has attracted widespread attention from both researchers and practitioners.

Previous internet marketing studies have shown that environmental cues can affect consumers’ purchase behavior *via* their perceptions and emotions (Loureiro and Roschk, 2014). Live streaming establishes a highly immersive connection between broadcasters and consumers by establishing daily social scenes and scene clues (Shen, 2012; Li et al., 2022). When consumers experience a stronger sense of social existence, the relationship between broadcasters and consumers is more interactive. Therefore, the on-site atmosphere is also improved with the aim of encouraging consumers to engage in purchase behavior (Lin, 2007). Simultaneously, better online stickiness reduces the cost of fan management (Wang et al., 2021). The cost of retaining loyal fans and potential consumers is lower than that of attracting new fans. Most of the extant research on the live streaming economy has focused on live broadcast platforms, live broadcast rewards, and audiences.

Previous studies have mainly focused on the perspective of businessmen and consumer behavior patterns (Wongkitrungrueng et al., 2020). In this context, with respect to the ongoing development of live streaming media, there are many cases of failure of live streaming media for personal reasons related to broadcasting companies, including incorrect explanations of the product by the broadcasters or situations in which the content introduced by the broadcaster is inconsistent with the actual product (Geng et al., 2022). However, the impact of broadcaster professionalism and the reputation of the platform on live streaming has not been the focus of previous research (Kang et al., 2021). Therefore, the joint influence of the professional knowledge of broadcasting platforms on the effect of live streaming media and consumer consumption behavior is worth investigating. In addition, the reputation of the platform plays a regulatory role in establishing consumer trust and increasing the effect of the broadcaster’s expertise, despite the fact that the role of platform reputation in live streaming has rarely been mentioned in previous studies.

To address these gaps in previous studies, this study proposes that the professionalism of broadcasters has a positive impact on consumer behavior in live broadcasts. In addition, consumers’ trust in the platform plays a mediating role between broadcaster expertise and consumers’ purchase intention. Moreover, the platform’s reputation moderates the relationship between broadcasters’ expertise and consumers’ trust in the platform. A live-streaming platform with a high reputation can make full use of the advantages of the strong professionalism of broadcasters. In the case of a platform with a poor reputation, even if the broadcaster is highly professional, it is difficult for consumers to establish trust with the broadcaster. In this way, when the reputation of the platform is high, this relationship is more stable. In addition, platform reputation moderates consumers’ trust in the platform as well as the relationship between broadcasters’ expertise and consumers’ purchase intention. The mediating effect is stronger when the platform’s reputation is higher. The questionnaire used in this study was distributed to 261 Chinese consumers. Data were analyzed to examine the research model and hypotheses.

By exploring the relationships among broadcasters, live streaming platforms, and consumers, this study contributes to different shareholders in different ways. For live broadcasters, this paper discusses the role of expertise in live streaming, promotes the transformation of broadcasters from simple broadcasters to social value creators, helps broadcasters cultivate an awareness of communication power, credibility, influence, and strengthens their expertise. For platforms, this study highlights the important role played by platform reputation in the live streaming effect. For consumers, this study helps consumers realize that the expertise of broadcasters and platform reputation can affect their consumption behaviors by affecting their sense of trust.

## Literature review and hypothesis development

The S-O-R (stimulus-organism-response) model was originally derived from environmental psychology (Mehrabian and Russell, 1974). This model has been used to study online environments, such as the internal organism responses of individuals on the internet.

“S” refers to the stimulation offered by an individual’s external environment; “O” refers to the changes in the individual’s internal state after being stimulated by the external environment, including cognitive and emotional aspects such as perceptions, attitudes, and experiences; and “R” refers to changes in the individual’s internal state after being stimulated by the external environment (Laato et al., 2020). Specifically, previous studies have focused on online communities, e-commerce, online education, and other fields (Dong and Li, 2018). In this context, “S,” the stimulus, includes interactivity and interaction, the environmental atmosphere, and satisfaction; O, the state of the organism, includes perceived pleasantness, perceived value, and social presence; and “R,” the response of the organism, includes impulsive purchase behavior and purchase intention (Guo et al., 2021).

### The influence of broadcaster expertise on consumers’ purchase intention

Expertise refers to the areas in which a broadcaster can provide correct and valid knowledge or experience (Hafferty, 2006). Interactions with a professional broadcaster *via* live streaming can significantly influence the perceived pleasure of online consumers and further influence their behavior (Dehouche and Assarut, 2020). Compared to the traditional e-commerce model, live e-commerce enables real-time information exchange among all participants in live streaming (Hu and Chaudhry, 2020). Broadcasters help consumers obtain a variety of information by introducing products, areas of production, and sales in real time and satisfying consumers’ needs for information in the context of their shopping decisions by explaining the broadcasters’ own expertise regarding the products in question (Hu and Chaudhry, 2020).

Professional broadcasters convey high-quality information through interactions with consumers in the context of live streaming and transmit a large number of professional signals to consumers (Biswas et al., 2006). In addition, broadcasters explain the products being sold, which improves the effectiveness of live streaming to provide agricultural assistance. Whether the consumer absorbs the broadcaster’s information affects their willingness to buy, thereby impacting their desire to consume (Syvertsen, 2004). Specifically, the broadcaster’s greater familiar with the recommended product, i.e., the more knowledge of the product that he or she has, the easier it is to convey signals of his or her own understanding or experience to allow the audience feel to perceive professionalism of the broadcaster. The more strongly consumers are influenced by the information stimulation conveyed by the broadcaster, the easier it is for consumers to develop a sense of identity with the broadcasters and the products they introduce. This sense of identity increases consumers’ desire to buy and thus makes it easier for consumers to make purchase decisions. The source of consumers’ willingness to buy is not only the product itself but also the expertise of the broadcaster.

Therefore, we hypothesize the following:

*Hypothesis 1:* Broadcaster expertise is positively related to consumers’ purchase intention.

### The mediating role of consumer trust

When the expertise of the broadcaster as a source of information increases, the credibility of the broadcaster’s information output is enhanced, and consumer identification with the product is thus strengthened (Rai et al., 2021). In live streaming, the expertise of the information source refers to the ability of the broadcaster to provide accurate and detailed information based mainly on the broadcaster’s in-depth knowledge and understanding of the recommended products. The broadcaster’s expertise affects consumer willingness (Chen and Liao, 2022).

Broadcasters’ expertise is a critical factor affecting the credibility of word-of-mouth information (Hussain et al., 2018). Whether the information provided by the broadcaster is accurate in terms of expertise, usage, and experience and whether it is perceived as valuable by consumers has a crucial impact on the broadcaster’s credibility. The greater the expertise of the broadcaster is, the more professional signals are conveyed by live streaming, and the more comprehensive and professional the introduction of products is, the easier it is to cause consumers to believe in the broadcaster’s ability and to establish a sense of trust with the broadcaster.

The higher the expertise of the source according to the consumers’ perceptions, the greater the reliability of the information disseminated by the head, and the greater the influence on the consumer as a recipient of the data (Park and Kim, 2021). Expertise can effectively reduce the perceived risk of the product to the consumer. This influence may deepen consumers’ favorable perceptions of the live broadcast or the product and may thus accelerate their purchase decisions.

In agriculture-assisted live streaming, the broadcaster has a high level of expertise that enables him or her to increase consumers’ trust by providing professional explanations while live streaming, which may increase consumers’ willingness to consume (Xu et al., 2022).

Therefore, we hypothesize the following:

*Hypothesis 2*: Consumer trust mediates the relationship between broadcaster expertise and consumers’ purchase intention.

### The moderating role of platform reputation

Reputation, as an intangible asset of a company, can create market entry barriers and foster customer retention, thus increasing the company’s competitive advantage (Schwaiger, 2004). Reputation is widely disseminated across various stakeholders, which can reduce the information asymmetry encountered by different actors in the transaction and prevent the distortion of communication among parties to the transaction; it can also benefit various market participants such as customers, merchants, or service personnel by reducing the opacity of transactions (Kreps and Wilson, 1982). Corporate reputation has a positive effect on customers’ willingness to buy. A firm’s reputation indirectly determines customer expectations and influences their purchase intentions (Bianchi et al., 2019).

According to SOR theory, consumers’ purchasing behavior is caused by various stimuli resulting from physiological and psychological factors associated with the consumer’s body as well as external factors (Jacoby, 2002). Different factors stimulate consumers to generate motivation, and due to this motivation, consumers make decisions to purchase products (Dong et al., 2018). The credibility of the live streaming platform plays a crucial role in determining the extent to which consumers trust and accept sellers in the e-marketplace (Hong and Cho, 2011; Hollowell et al., 2019). In live streaming for agricultural assistance *via* broadcast platforms with high visibility, such as those featured in people’s daily agricultural aid broadcasts, it is easier for consumers to establish trust with the broadcaster when the professionalism of the broadcaster is exemplary, and when the broadcaster’s actions and language when introducing products are more professional, consumers are more likely to establish trust with the broadcaster based on these actions and this language, and they are thus more likely to trust the broadcaster’s introduction of the product, which is usually aimed at encouraging consumers to purchase the product; accordingly, this trust in turn promotes consumers’ behavior (Fu et al., 2022).

Conversely, it is difficult for broadcasters to convince consumers *via* platforms with low reputations (Wang et al., 2022). Even if the broadcasters in question are very professional and their explanations are very effective, due to the poor reputation of the broadcasting platform, consumers may feel as if it is difficult to attract good broadcasters to a platform with a poor reputation, and so even if the broadcaster is truly professional, it is difficult for consumers to develop trust in the broadcaster; consequently, it is difficult for consumers to complete their product purchase behavior.

Therefore, we hypothesize the following:

*Hypothesis 3*: Platform reputation moderates the relationship between broadcaster expertise and consumer trust such that this relationship is stronger when platform reputation is higher.

Trust in merchants is widely known to reduce the perceived risk of transactions. In live streaming pertaining to goods, consumers are willing to purchase products from a well-known live streaming platform because they trust the institutional mechanism associated with the famous live streaming platform. According to SOR theory, consumers’ consumption behavior is induced by stimuli; even though consumers do not know the broadcaster, it is easier for consumers to establish a sense of trust and to enhance their consumption aspirations if they find that the live streaming is hosted by a famous platform. In live streaming platforms with a good reputation, when the broadcaster provides many professional product demonstrations, this situation provides consumers with a large amount of reliable professional information, which leads to consumers’ recognition of the broadcaster, increases their trust, and ultimately results in consumers’ online purchase behavior. This situation provides consumers with a wealth of reliable and specialized information, which leads to consumer identification with the broadcaster and increases their trust, thus promoting online purchase behavior (Dong et al., 2017; Osei-Frimpong et al., 2019).

Therefore, we hypothesize the following:

*Hypothesis 4:* Platform reputation moderates the mediating effect of consumer trust in the platform on the relationship between broadcaster expertise and consumers’ purchase intention such that this mediating effect is stronger when platform reputation is higher.

**FIGURE 1:**
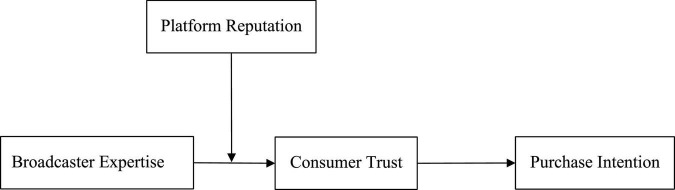
Theoretical model in this study.

Figure 1 displays the theoretical framework of this study.

## Materials and methods

### Sample and procedures

In this study, we adopted a questionnaire survey method using the snowball sampling method based on personal relationships. Three research assistants for this project all frequently watched farmer-assisted live streaming and initiated the sampling process by consulting their personal contacts. These research assistants helped us distribute our questionnaire survey to encourage further participation. This method has been widely used, and it is effective for collecting data (Geng et al., 2022). All responses were voluntary and anonymous, and all respondents had experience watching live broadcasts aimed at assisting farmers. We assured respondents that the data would be used only for research purposes.

We invited participants to respond to our questionnaire, which assessed their opinions of broadcasters’ expertise, consumers’ trust, platform reputation, and purchase intention and collected demographic variables.

A total of 261 questionnaires were distributed, and all usable surveys were returned. To ensure the reliability of the data sources, we collected data concerning participants’ gender, age, education, and monthly salary. The effective sample included 55.7% male and 44.3% female participants. The average age of participants was 30.2 years old. In terms of salary, 32.0% of participants’ salaries were less than 5,000 RMB per month, and the percentages of participants’ salaries in the 5,000–1,0000 and 10,000–20,000 ranges were both 30.4%. In addition, 7.1% of the participants’ salaries were more than 20,000 per month. In addition, participants had different levels of education. The three levels of 3-year college or below, bachelor’s degree and master’s degree or above accounted for 31, 38.7, and 30.3% of the sample, respectively.

### Measurements

#### Broadcaster expertise

Broadcasters’ expertise was measured using the scale developed by Ohanian (1990), and participants responded to items on a 5-point Likert scale ranging from 1 (very low) to 5 (very high). Four items were included in this scale, and a sample item was “I think the broadcaster of this live broadcast has professional skills…” The Cronbach’s alpha coefficient for this scale was 0.75.

#### Consumer trust

Consumer trust was measured using a 3-item scale adapted from Kim et al. (2015). This scale assessed consumers’ trust; a sample item was “This platform gives the impression that it keeps promises and commitments.” Participants responded on a 5-point Likert scale ranging from 1 (strongly disagree) to 5 (strongly agree). The Cronbach’s alpha coefficient for this scale was 0.82.

#### Platform reputation

Three items adapted from Keh and Xie (2009) were used to assess platform reputation. Participants responded on a 5-point Likert scale ranging from 1 (strongly disagree) to 5 (strongly agree). A sample item was “The focal platform is a highly regarded platform.” The Cronbach’s alpha coefficient for this measure was 0.81.

#### Purchase intention

We rated participants’ purchase intention based on the suggestions of Martins et al. (2019). We listed three items: “I find purchasing this product/service to be worthwhile,” “I will frequently purchase this product/service in the future,” and “I will strongly recommend purchasing this product/service to others.” Participants responded on a 5-point Likert scale ranging from 1 (strongly disagree) to 5 (strongly agree). The Cronbach’s alpha coefficient for this measure was 0.87.

#### Control variables

Demographic information was collected during the study to ensure the minimal impact of exogenous variables. These control variables included participants’ gender, age, salary, and level of education.

## Results

### Descriptive statistics

Table 1 presents descriptive statistics and correlations among the study variables used in this study. As shown in Table 1, broadcasters’ expertise was positively related to consumer trust (*r* = 0.14, *p* < 0.05) and purchase intention (*r* = 0.15, *p* < 0.05). In addition, platform reputation had a positively significant relationship with purchase intention (*r* = 0.33, *p* < 0.05). Taken together, these results provide preliminary support for our hypotheses.

**TABLE 1:** Descriptive statistics and correlations.

	Mean	*SD*	1	2	3	4	5	6	7	8
1. Expertise	3.67	1.04	−							
2. Consumer trust	3.49	1.57	0.14*	−						
3. Platform reputation	5.28	1.45	−0.25**	0.24**	−					
4. Purchase intention	3.67	1.61	0.15*	0.42**	0.33**	−				
5. Gender	0.44	0.50	0.06	–0.05	–0.01	–0.12	−			
6. Age	30.16	7.25	–0.04	–0.05	0.14*	–0.07	−0.13*	−		
7. Salary	2.13	0.95	0.24**	–0.03	−0.28**	–0.02	–0.01	−0.18**	−	
8. Education	1.99	0.78	0.14*	–0.04	–0.06	–0.10	–0.04	–0.06	–0.02	–

*N* = 261.

Gender: 0 = male, 1 = female.

Education: 1 = 3-year college or below, 2 = bachelor, 3 = master or above.

*p < 0.05; **p < 0.01.

### Hypothesis testing

To examine the validity of our measurements, we conducted exploratory factor analyses (EFAs). Four constructs emerged, and each item was loaded on the intended dimension. The results of the EFAs can be found in Table 2.

**TABLE 2:** Results of exploratory factor analyses.

	Factor 1	Factor 2	Factor 3	Factor 4
Expertise 1	0.75			
Expertise 2	0.77			
Expertise 3	0.64			
Expertise 4	0.81			
Trust 1		0.84		
Trust 2		0.83		
Trust 3		0.83		
Reputation 1			0.85	
Reputation 2			0.84	
Reputation 3			0.79	
Intention 1				0.84
Intention 2				0.90
Intention 3				0.81

Extraction method: Principal component analysis.

Rotation method: Varimax with kaiser normalization.

Small coefficients (lower than 0.3) were suppressed.

To test our hypotheses in further detail, we used the multiple regression approach to examine Hypotheses 1 and 2 in accordance with the suggestions of Baron and Kenny (1986). The results are presented in Table 2. Hypothesis 1 proposed that broadcaster expertise is positively related to consumers’ purchase intention. In Model 5, broadcaster expertise was positively related to consumers’ purchase intention (β = 0.20, *p* < 0.01). Thus, Hypothesis 1 was supported.

Hypothesis 2 proposed that consumer trust mediates the relationship between broadcasters’ expertise and consumers’ purchase intention. Model 6 revealed that broadcasters’ expertise was positively related to consumers’ purchases (β = 0.14, *p* < 0.05). Consumers’ trust was also positively related to consumers’ purchases (β = 0.40, *p* < 0.01). Moreover, the coefficient of broadcasters’ expertise for purchase intention decreased from 0.20 to 0.14, indicating that consumers’ trust plays a partial mediating role in the relationship between broadcasters’ expertise and purchase intention. We used the Process macro (Model 4; Hayes, 2013) to further validate this mediating effect. The 95% confidence interval of the indirect effect was (LLCI = 0.01, ULCL = 0.21) and did not contain zero. Thus, Hypothesis 2 was supported.

**FIGURE 2:**
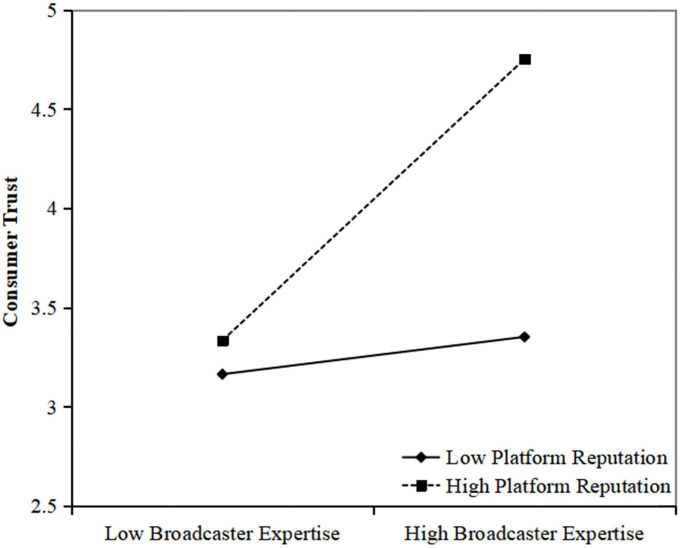
Interaction of broadcaster expertise and platform reputation on consumer trust.

Hypothesis 3 proposed that platform reputation moderates the relationship between broadcasters’ expertise and consumer trust such that this relationship is stronger when platform reputation is higher. Accordingly, based on the suggestions of Aiken and West (1991), we used the multiple regression approach to examine Hypothesis 3. As shown in Table 3, Model 3 revealed that the interaction item significantly and positively predicted the relationship between broadcaster expertise and consumer trust (β = 0.23, *p* < 0.01). We further conducted a simple slope analysis. As shown in Figure 2, broadcaster expertise was significantly related to consumer trust (*b* = 0.68, *t* = 5.05, *p* < 0.01) when platform reputation was high but non-significant (*b* = 0.09, *t* = 0.79, n.s.) when platform reputation was low. Thus, Hypothesis 3 was supported.

**TABLE 3:** Results of hierarchical multiple regression.

Variables	Consumer trust			Purchase intention		
	Model 1	Model 2	Model 3	Model 4	Model 5	Model 6
**Control variables**						
Gender	–0.07	–0.08	–0.06	−0.14*	−0.15*	−0.12*
Age	–0.07	–0.10	–0.11	–0.11	–0.11	–0.08
Salary	–0.04	–0.02	–0.02	–0.04	–0.09	–0.06
Education	–0.05	–0.06	–0.04	−0.13*	−0.16*	−0.13*
**Independent variable**						
Broadcaster expertise		0.22**	0.26**		0.20**	0.14*
**Mediator**						
Consumer trust						0.40**
**Moderator**						
Platform reputation		0.31**	0.25**			
**Interaction**						
Broadcaster expertise × consumer trust			0.23**			

*R* ^2^	0.01	0.12	0.16	0.04	0.08	0.23
Δ*F*	0.62	15.01	13.01	2.53	10.00	48.62

*N* = 261.

*p < 0.05; **p < 0.01.

The regression coefficients in the table are all standardized regression coefficients.

Hypothesis 4 predicted that platform reputation moderates the mediating effect of consumers’ trust in the platform on the relationship between broadcasters’ expertise and consumers’ purchase intention such that this mediating effect is stronger when platform reputation is higher. According to moderator values set at the mean as well as at plus/minus one *SD* from the mean, different degrees of platform reputation indicating low, middle and high levels of such reputation were distinguished. The conditional indirect effect of platform reputation on broadcaster expertise and consumers’ purchase intention *via* consumer trust are shown at the bottom of Table 4.

**TABLE 4:** Moderated mediation analysis examining the impact of platform reputation on the relationship between broadcasters’ expertise and consumers’ purchase intention with consumers’ trust as a moderator.

Predictor	β	SE	*t*	LLCI	ULCI
**Outcome: Consumer trust**					
Constant	4.60	0.58	7.90**	3.45	5.74
Broadcaster expertise	0.38	0.09	4.06**	0.20	0.57
Platform reputation	0.27	0.07	3.98**	0.14	0.41
Broadcaster expertise × platform reputation	0.20	0.06	3.61**	0.09	0.32
**Outcome: Purchase intention**					
Constant	3.66	0.63	5.75**	2.41	4.91
Broadcaster expertise	0.22	0.09	2.34*	0.03	0.40
Consumer trust	0.41	0.06	6.97**	0.29	0.53
**Conditional bootstrap estimates for consumer trust**					

**Platform reputation**	**Effect**	**Boot SE**	**Boot LLCI**	**Boot ULCI**	

–1 *SD*	0.04	0.05	–0.05	0.13	
Mean	0.16	0.05	0.06	0.27	
+1 *SD*	0.28	0.08	0.14	0.44	

Bootstrap sample size = 5000.

The level of confidence for all confidence intervals is 95%.

LL, Lower limit; CI, Confidence interval; UL, Upper limit.

*N* = 261.

*p < 0.05; **p < 0.01.

The results indicated that for low platform reputation, consumer trust did not play a moderating role in this context. The 95% confidence interval of the bootstrap was (LLCI = –0.05, ULCL = 0.13), which contained zero. For medium and high levels of platform reputation, the mediating effects of consumer trust were significant, and the 95% confidence intervals were (LLCI = 0.06, ULCL = 0.27) and (LLCI = 0.14, ULCL = 0.44), which did not contain zero. In addition, the index of the moderated mediation was 0.08, and the 95% confidence interval did not contain zero (LLCI = 0.04, ULCL = 0.13). Based on these results, Hypothesis 4 was supported.

## Discussion

### Theoretical contributions

Our research makes three theoretical contributions. First, against the social backdrop of the booming live broadcasting economy and due to the severe impact of COVID-19 on the agricultural economy, farmer-assisted live streaming emphasizes a variety of products and full chain marketing. This practice also includes shareholders such as the government, live broadcasting platforms, consumers, and farmers. Direct broadcasting regarding agricultural products has played an essential role in developing society as a whole, especially with regard to rural agriculture and farming. However, in the field of marketing, most previous research has focused on the short-video economy (Kang et al., 2021). Accordingly, the role of farmer-assisted live streaming, which has its own specific background in China, remains underexplored. Therefore, our research enriches the literature on this topic.

In addition, this study introduces broadcasters’ expertise into the relevant framework. Previous research on broadcasters’ characteristics has mainly focused on the field of news communication, primarily in terms of broadcasters’ voices and emotional management (Wongkitrungrueng et al., 2020). Based on broadcasters’ expertise, this study emphasizes the impact of broadcasters’ live streaming experiences, understanding of products, and relevant knowledge regarding agricultural live broadcasts. In addition, this study also highlights the mediating effect of consumer trust in this context, revealing the trust mechanism operative in the relationship between broadcaster expertise and consumer purchase intention. The more expertise that a broadcaster demonstrates in the context of live streaming, the more professional signals he or she conveys, which cause consumers to trust the broadcaster and promote their purchase intention.

Moreover, the role of platform reputation is underexplored in the previous literature (Lu and Chen, 2021). This study combines the current situations of different levels of broadcasters in the context of agricultural-assisted live broadcasting with the impact of live broadcasting platforms with different reputations on consumer behavior. For example, well-known platforms or platforms with poor reputations have different effects on consumer trust and broadcaster professionalism in the context of agricultural-assisted live broadcasting. Given the important role played by broadcasters with respect to the effect of live streaming and the importance of the platforms’ reputation in this context, we use platform reputation as a moderator. Our results demonstrate the moderating relationship between professional expertise and consumer trust. This finding shows that establishing trust between the audience and the broadcaster may be key to the promotion of consumers’ behavior in the context of agricultural-assisted live streaming. In addition, to establish a sense of trust more effectively, choosing a live broadcast platform with a good reputation is essential.

### Practical implications

This research explores the factors that influence consumers’ propensity to make a purchase in the context of farmer-assisted live streaming based on a survey distributed using the snowball sampling method. Broadcasters, consumers, and live streaming platforms are the three most important elements in farmer-assisted live streaming. Our findings have significant implications for businesses, customers, society, and live streaming platforms. These results can provide ideas for expanding marketing channels for agricultural products, which can help reduce poverty and provide assistance to farmers.

First, this research indicates that broadcasters’ expertise is one of the most important elements influencing the reliability of the material of online broadcasts. The majority of the diverse information that consumers receive relies on broadcasters’ explanatory presentations. In live streaming, the professionalism of the information source refers to the ability of the broadcaster to obtain insight into the recommended product and deliver accurate, complete information (Chen and Liao, 2022). The familiar and knowledgeable consumer’ are with regard to the product, its area of production, and its distribution, the more likely it is for the consumer to purchase the product (Xu et al., 2022). Therefore, it is essential that broadcasters receive training to enhance their professional expertise and their familiarity with the complete online broadcasting supply chain.

Second, this research demonstrates that a platform’s good reputation moderates and strengthens the relationship between broadcasters’ expertise and consumer trust. This factor reduces the perceived level of risk connected with the transaction. In regard to live streaming, people are more likely to acquire products from well-known platforms. Therefore, to effectively aid farmers in producing income and reducing poverty, the event organizer should choose a larger, more reputable live streaming network.

Finally, consumers’ trust is key to the success of farmer-assisted live streaming. According to Yoon (2002), internet transactions rely on consumer trust and are virtual in nature (faceless and storeless); hence, the trust mechanism operative in this context merits considerable consideration. Consumers’ faith in agricultural products can be bolstered in a variety of ways, including by enhancing the protection of consumers’ purchasing security and privacy, maximizing customer service, expanding brand visibility, demonstrating activity on social media platforms, and raising awareness.

### Limitations and directions for future research

This research faces several limitations. First, the scope of the present study focuses on agricultural products in the current Chinese environment. There is no comprehensive investigation of whether the inclusion of various types of products in various types of live streaming affects consumers’ purchasing intentions. The applicability of the conclusions of this research to other universal definitions of live streaming or to consumers in other cultures requires additional analysis. Due to cultural differences, purchase experiences and consumer behaviors on Western e-commerce platforms differ dramatically from those observed in China. Consequently, consumer trust and purchase intents for live streaming products vary across cultures, which exerts an influence in this context.

Second, the current study focuses primarily on the joint role of broadcasters and platforms. However, future research could broaden the current study to investigate other potential contributing elements, such as the eloquence of the broadcaster, their sense of humor, platform bonuses, and the impact of diverse areas and cultures on live streaming data. Additionally, buyers may have different purchase intentions for different live streaming items. This study did not examine the effect of various live streaming products. Future studies might therefore concentrate on this area and evaluate consumer trust and purchase intent across various live streaming products.

Third, the use of questionnaires as a research tool in this study provides only limited evidence to support causal inferences and does not totally eliminate the inherent bias of conventional research methodologies. In addition, although a scenario-based questionnaire is used in this study to gauge consumer purchase intentions, such intentions may not necessarily transfer into actual purchase behavior. To collect more extensive and accurate data, future investigations could utilize a combination of experiments and longitudinal surveys.

Finally, this research focuses on the effect of broadcaster professionalism in farmer-assisted live streaming and platform reputation on consumer stickiness. Future research could investigate additional or alternative variables that influence the relationship between other parameters and purchase intentions or other dependent variables. For example, individuals’ emotions or stress may influence their behaviors (Wang and Li, 2017). Therefore, subsequent studies on live streaming may lead to different results due to variations in the independent variables, even if the dependent variables remain constant. In reality, elements such as the live streaming platform’s popularity and the live streaming environment vary across many types of live streaming for farmers. In future studies, control variables could potentially contain and combine these elements for targeted research.

## Data availability statement

The raw data supporting the conclusions of this article will be made available by the authors, without undue reservation.

## Ethics statement

Ethical review and approval was not required for the study on human participants in accordance with the local legislation and institutional requirements. The patients/participants provided their written informed consent to participate in this study.

## Author contributions

JLi developed the theoretical framework and worked on the literature review and manuscript writing. RZ developed the theoretical framework and worked on the manuscript writing. HS and WM worked on the manuscript writing. HS and JLu worked on data collection and analysis. All authors contributed to the article and approved the submitted version.
